# Draft Genome Sequence of the Probiotic Yeast *Kluyveromyces marxianus fragilis* B0399

**DOI:** 10.1128/genomeA.00923-16

**Published:** 2016-09-01

**Authors:** Sara Quarella, Paola Lovrovich, Simone Scalabrin, Ilenia Campedelli, Ana Backovic, Veronica Gatto, Federica Cattonaro, Alessandro Turello, Sandra Torriani, Giovanna E. Felis

**Affiliations:** a Department of Biotechnology, University of Verona, Verona, Italy; b Laboratori Turval S.r.l, Udine, Italy; c IGA Technology Services S.r.l., Udine, Italy

## Abstract

Here, we report the draft genome sequence of *Kluyveromyces marxianus fragilis* B0399, the first yeast approved as a probiotic for human consumption not belonging to the genus *Saccharomyces*. The genome is composed of 8 chromosomes, with a total size of 11.44 Mb, including mitochondrial DNA.

## GENOME ANNOUNCEMENT

The probiotic lactic yeast *Kluyveromyces marxianus fragilis* B0399 (TURVAL B0399) is a strain belonging to a species naturally occurring in kefir and cheese, which is isolated from whey and curds of cow milk. It is the first yeast not belonging to the genus *Saccharomyces* with probiotic activity that has appeared in the market, and it was approved as a probiotic for both animal feeding (1) and human consumption (2). This strain was shown to remain viable after consumption, surpassing the gastric barrier and colonizing the gut adhering to the enterocytes of the intestinal epithelium (3). Moreover, it regulates intestinal activity during antibiotic therapy, thanks to the yeast intrinsic resistance to bacterium-targeting antibiotics (4). Furthermore, its β-galactosidase activity, absent in strains of the genus *Saccharomyces*, permits the degradation of lactose, generating glucose and galactose; therefore, it is useful in lowering the effects of lactose intolerance in susceptible individuals (5). Several *in vitro* (3) and *in vivo* (4, 6) experimental evidences demonstrated that *K. marxianus* B0399 is able to modulate the immune response, reducing the proinflammatory cytokine levels, thus being useful for mitigation of the effect of several diseases, such as irritable bowel syndrome (6, 7).

Sequencing of its genome was performed to gain more detailed information on its characteristics as a probiotic and to broaden knowledge on the genomic biodiversity in the species *K. marxianus*, since other strains whose genome sequences are available are used for bioethanol production or other applications (8–10).

Microbial DNA, isolated using the Wizard Genomic DNA purification kit (Promega), was used to construct three libraries with insert sizes of 578 nucleotides (nt), 626 nt, and 5 kb, which were amplified and sequenced using the Illumina HiSeq 2000 at IGA Technology Services (Udine, Italy), producing clean data containing 20 million paired reads with 150 nt at 270-fold coverage.

Trimmed reads were *de novo* assembled using AllPaths-LG version 48359 (11) with default parameters, except for TARGETS = Std., HAPLOIDIFY = T, CLOSE_UNIPATH_GAPS=F, DRY_RUN=F, MIN_CONTIG=500, and MAX_MEMORY_GB=120. The contigs were scaffolded using IMR/DENOM version 0.4.0f (12) and ordered by ScaPA release 3 (IGA Technology Services) using the genome of *K. marxianus* NBRC1777 (GenBank accession numbers AP014599 to AP014607) as a reference.

The genome sequence of B0399 was 11.44 Mb, and the G+C content was 40%. The genome was composed of eight chromosomes, including mitochondrial DNA. The assembly generated 109 scaffolds, with a maximum length of 1.3 Mb and an *N*_50_ length of 743,117 bases. A total of 5,373 putative proteins of *K. marxianus* B0399 were predicted using tBLASTn analysis against the reference genome. The key enzyme for lactose degradation (β-galactosidase) was highly similar to the corresponding enzyme from *Kluyveromyces lactis*, confirming that this yeast can be successfully used for the reduction of the effects of lactose intolerance in susceptible individuals.

### Accession number(s).

This whole-genome shotgun project has been deposited at DDBJ/ENA/GenBank under the accession no. LXZY00000000. The version described in this paper is the first version.
